# Analysis of Volatile Organic Compounds from Compost

**DOI:** 10.3390/atmos16050591

**Published:** 2025-05-14

**Authors:** Shastine K. Berger, Rosario C. Morales, Katherine McCown, Kylie Wilson, B. Thomas Jobson, Nancy A.C. Johnston

**Affiliations:** 1 Physical, Life, Movement, & Sport Sciences Division, Lewis-Clark State College, Lewiston, Idaho; 2 Department of Civil and Environmental Engineering, Washington State University, Pullman, Washington

**Keywords:** Compost, volatile organic compounds, chromatography, air sampling, food waste, yard waste

## Abstract

Many US states have adopted regulations to divert food waste from landfills to composts. While this may lower greenhouse emissions from landfills, volatile organic compound (VOC) emissions from compost may contain hazardous air pollutants or produce odors, posing potential public health concerns. Effective methods to analyze speciated VOCs in compost are needed to better understand VOC source generation. Here, a two-component compost sampling method was developed and employed consisting of a chilled impinger and pump apparatus to trap water-soluble VOCs, and dual sorbent tubes to capture hydrophobic VOCs in yard and food/yard waste compost. VOCs were measured via headspace gas-chromatography with flame ionization detection (HS-GC-FID) and thermal desorption-gas chromatography-mass spectrometry (TD-GC-MS). Overall, there was higher VOC generation within higher temperature compost piles, with concentrations ranging up to 27,000 ppm for ethanol and 3,500 ppm for methanol. Alpha-Pinene and D-Limonene were seen in these piles with concentrations over 1600 ppb. Methanol and ethanol were more than one thousand times as concentrated in mixed food/yard waste than yard waste alone, while terpenes were seen in slightly higher concentrations for yard waste than the mixed food/yard waste. Methanol was observed higher than permissible indoor levels and may pose potential health risks.

## 1. Introduction

Compost is used vastly within the agricultural industry where it acts as fertilizer and soil amendments [1]. It is a more efficient way to decompose organic waste due to its aerobic properties and is growing in usage [2]. Many municipal facilities are turning to composting due to the aerobic decomposition present in comparison to anaerobic decomposition found in landfills, where methane gas emissions are found [3]. Greenhouse gases, including methane and carbon dioxide are emissions of concern for landfills and composting due to their influence on global warming [4]. Multiple US states have banned commercial food waste from landfills, to help reduce waste as well as greenhouse gases, with limited success [5]. Other strategies to reduce greenhouse gas emissions include the use of livestock manure [6], biofilters [7], and microbes [8]. Similarly, methods have been utilized to determine factors affecting emissions from compost and their potential for ozone (smog) formation [9]. There have been a variety of studies determining the major gas emissions at landfill and compost facilities, most of which are emitted from landfills [10–11]. The difference in emissions between the facilities is due to the different types of decomposition processes [12]. As previously mentioned, compost facilities use aerobic decomposition while landfills rely on anaerobic decomposition [10]. Greenhouse gases are known to come from compost due to the degradative process it goes through [13]; however, there are a variety of other compounds that are emitted from compost, such as volatile organic compounds (VOCs) [14].

Disadvantages of composting include production of odorous emissions of VOCs [15–17]. VOCs are carbon containing compounds with a range of volatility, or their ability to evaporate from the liquid to gas phase. VOC emissions are influenced by a variety of factors including moisture levels, oxygen content, compost composition, and temperature [18]. Some VOCs are classified as air toxics by the US Environmental Protection Agency (EPA) [19], and may cause possible health risk to humans or environmental damage in high enough concentrations [11,20]. The US Occupational Safety and Health Administration (OSHA) regulates work-related chemical exposures, setting permissible exposure limits (PEL) for several air pollutants [21]. Due to environmental and health concerns, several municipalities have regulations on VOC emissions from compost facilities, or the facilities require air permitting. In a recent review of the field by Nordahl et al. [22], VOC emission factors from compost were reported to range from 6 × 10^−5^ to 1.7 × 10^−3^ kg VOC/kg wet feedstock. However, there is still a general lack of data on speciated VOC emissions from compost, due to the time consuming and expensive sampling and analysis protocols.

Some methods commonly used to study compost emissions include simulation of compost facilities using digestion systems [23] and environmental flux chambers [9]. The digestion chambers use a filtration system for air entering the compost chamber, and samples collected via canisters or charcoal/sorbent tubes [23, 24]. Environmental flux chambers are placed directly on top of compost piles with different aeration patterns, analyzing total VOC emissions in a given compound group such as alcohols, acids, and hydrocarbons [9]. After collection of the VOCs, gas chromatography, mass spectrometry and greenhouse gas analyzers are typically used to identify VOCs and greenhouse gas composition [9,13,25–26]. One problematic issue with these methods is their lack of tolerance for humid compost environments. A method developed to overcome this is described by South Coast Air Quality Management District (SCAQMD) Method 25.3, which uses a water impinger to capture moisture and water-soluble VOCs while hydrocarbons like monoterpenes are captured in a SUMMA canister for analysis [27]. The analysis of water-soluble VOCs by this method is often omitted, as the focus is the total VOCs collected after the impinger water collection stage. A more comprehensive method which allows for the measurement of both water-soluble and insoluble VOCs would be useful.

The purpose of this study was two-fold. The first goal was to develop a dual sampling and instrumental method to measure speciated VOCs in compost. The second goal was to compare compost VOC concentrations from two different sources, yard waste and mixed food/yard waste. The technique used a water impinger to remove humidity from the sampling line by capturing water-soluble VOCs. The impinger was coupled with a sorbent tube to capture the remaining water-insoluble VOCs. Two chromatographic methods were used (headspace-gas chromatography and thermal desorption-gas chromatography-mass spectrometry) to analyze VOCs captured from compost. Yard waste and mixed food/yard waste compost piles were constructed. Several variables were measured when sampling from the piles including: temperature, age, and composition. 119 VOCs were analyzed and captured during the aging process of controlled compost piles, including alcohols, monoterpenes, sulfides, and hydrocarbons.

## 2. Materials and Methods

### 2.1. Overview

Compost piles were built specifically for research purposes at the Washington State University Compost Facility (WSU CF). Two main composition types (yard waste and mixed food/yard waste) were sampled in duplicate for up to 21 days, totaling 90 samples. Soil probes were inserted in the compost pile to withdraw air through a water impinger and then through a sorbent tube. The water and air samples were analyzed via headspace gas-chromatography (HS-GC) and thermal desorption-gas chromatography-mass spectroscopy (TD-GC-MS), respectively. This method of sampling and analysis was tested for efficacy and to characterize compost at the WSU CF.

### 2.2. Compost Setup

Over the course of two separate compost trials, 19 July 2022 – 22 July 2022 and 31 August 2022 – 14 September 2022, different compost compositions were analyzed. The compost material makeup included food plus yard waste (FY) in trial B, while trial A consisted of strictly yard waste (YW) (Table 1). During the analysis of the different compost piles, temperatures were monitored. Pile 1 was a controlled pile with lower temperatures and Pile 2 had naturally higher temperatures. The piles were controlled by the addition of insulation (a tarp) to prevent heat from escaping.

### 2.3. Sampling Design

Two different types of samples were taken via pumped air from the compost pile; condensing of water-soluble compounds using an impinger, and subsequent collection of water-insoluble compounds using a sorbent tube. The water impinger system contained 5.00 mL of deionized water pipetted into a 10.00 mL headspace vial, nested inside a Savillex Teflon impinger. Markes Tenax^®^TA-Sulficarb dual sorbent tubes (3.5”/89 mm length, ¼”/6.4 mm outer diameter and 5 mm inner diameter, stainless steel, inert coated) were utilized for sampling via adsorption.

**Figure 1:**
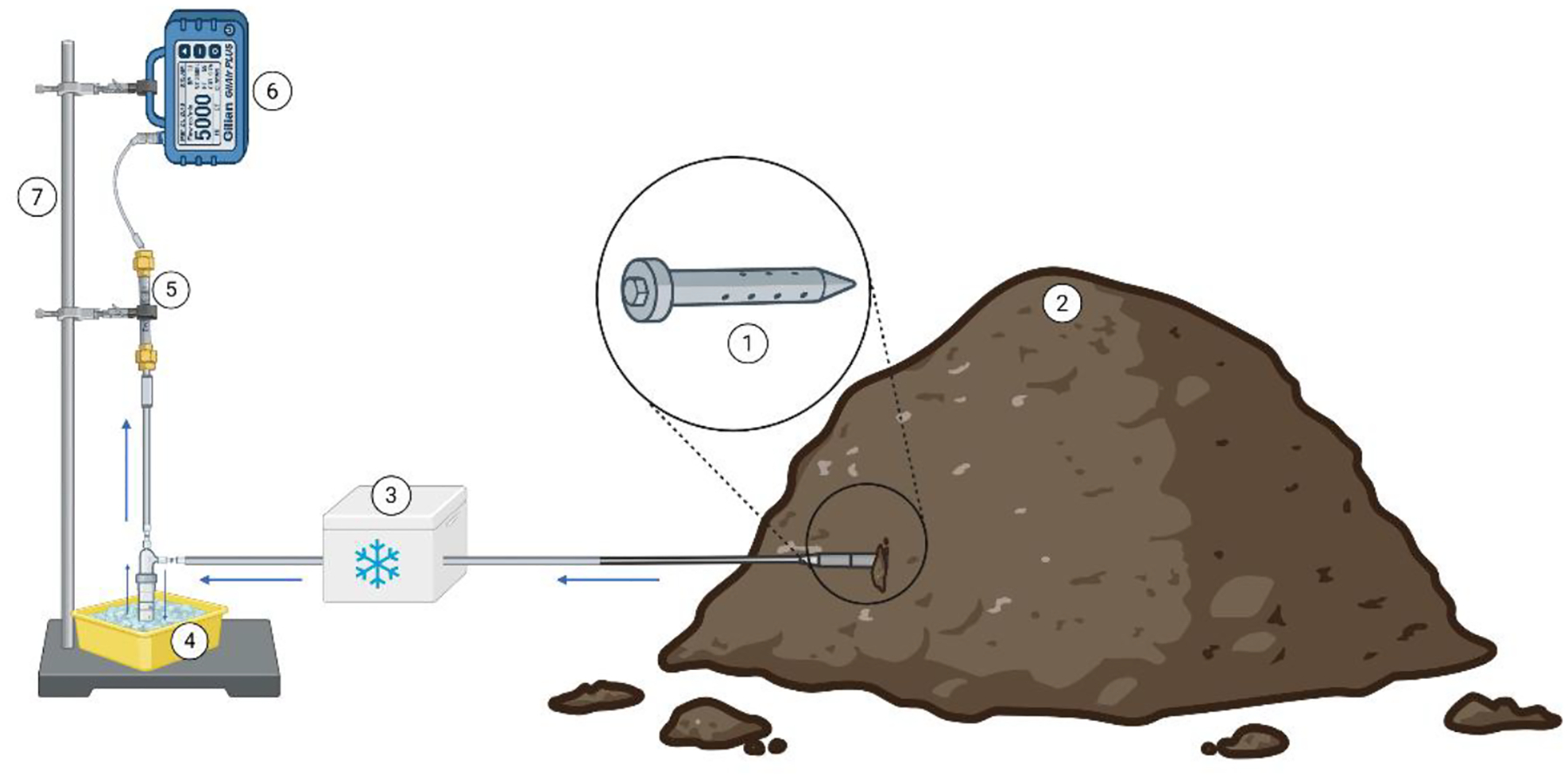
Figure 1. Schematic of the compost sampling setup used for simultaneous collection of air and water-soluble analytes. (1) Stainless steel soil probe inserted into the (2) compost pile enables sampling of emitted volatiles. The air is pulled sequentially through (3) a cooler containing ice, (4) a 10 mL collection tube housed within a Teflon impinger submerged in an ice bath (for water-soluble compounds), and (5) a dual bed sorbent tube with Tenax-TA and Sulficarb (for water insoluble compounds), while being drawn into the (6) Gilian^™^ air sampling pump. Components 1–6 are connected via Teflon tubing, with blue arrows indicating direction of airflow. A support stand (7) secures the air pump and sorbent tube in place throughout the sampling process. Created in https://BioRender.com

Teflon tubing (¼-½” outer diameter) attached to a Gillian Air pump connected both the sorbent tube and the impinger to the compost pile or sampler. A Markes Soil Probe (12”/304.8 mm length) was used with some samples, while the other samples contained a Teflon tube placed directly in the compost pile. Figure 1 shows the sampling apparatus.

Samples were collected within compost piles built in the summer/fall of 2022 at the WSU CF in Pullman, WA. The focus was on two piles built for the concurrent research conducted to compare the different factors that affect VOC emissions from compost (Table 1). Two locations (top and side of each pile) were analyzed in duplicate, in both yard waste (YW) compost (n=40) and mixed food/yard (FY) waste (n=50) [28]. Samples were taken beginning on day five and repeated at various increments after a new pile was made. Details on the compost piles are in Table 1. The side compost location had a ½” Teflon tube that was inserted four feet into the side of the pile with a ½”/12.7 mm to ⅛”/3.18 mm adapter to access the middle of the pile. A total of 0.5–2.6 L of air was bubbled into the water samples and 0.05–0.59 L into the air samples. When the sample location was not in use, copper tubing was inserted in the Teflon tubing to prevent the tube from collapsing due to the high temperatures of the compost pile. The top compost location had a soil probe that was inserted one foot into the top of the pile after the matured layer was scraped off. While sampling occurred, a variety of field data was recorded including; weather conditions, time of sampling, location on pile, flow rate, sample duration, temperature of the pile, and pile reference number. The experiments were repeated using compost of yard waste and mixed food/yard waste.

### 2.4. Analytical Protocols

Samples were capped and transported back to the lab. A thermal desorption-gas chromatography-mass spectrometer (TD-GC-MS) was used to analyze the air component and a headspace gas-chromatograph with flame ionization detection (HS-GC-FID) was used to analyze the water component. Prior to sampling, the sorbent tubes were conditioned in a heated oven (100°C for 30 minutes, 200°C for 75 minutes) with 100 mL/min nitrogen flow through the tube. The sorbent tubes were blanked using TD-GC-MS with the same method used for analysis. The minimal residue left on the blank tubes were subtracted (average of blanks) from the respective amounts of VOCs measured.

The aqueous samples required the addition of 0.50 grams of sodium sulfate to release the trapped gases back into the headspace of the vials. These samples were then analyzed with the HS-GC-FID. The sorbent tubes were purged with nitrogen (100 mL/min for 6 minutes) after sampling then analyzed with TD-GC-MS with method adapted from United States Environmental Protection Agency Method TO-17 [29] and summarized by Scott et al. [30] and Dickinson et al. [31]. Instrumentation parameters are listed in Table 2 and further chromatographic details in Tables S1 and S2.

### 2.5. Calibration

To verify accuracy and to adjust for instrument drift, each instrument was initially calibrated with five levels, and upon each sample batch analysis. If a calibration check was outside of the 80–120% recovery range, the system was recalibrated. HS-GC-FID standards were created using pure liquid VOCs (Fisher) that were diluted with deionized water in volumetric proportions, with concentrations ranging from 0.2 μL/L to 200 μL/L. These mixtures (5 mL) were then analyzed by the HS-GC-FID after the addition of 0.50 grams of sodium sulfate in a 10-mL headspace vial. Similarly, liquid standards for the TD-GC-MS were created using 100 μL/L in methanol and 0.5 μL injection, resulting in 0.7–30 ng VOC spiked into the sorbent tube. In addition, prepared standard mixes were purchased and utilized: Airgas TO-65 Component Mix 1ppm in nitrogen, Airgas Ozone Precursor/PAMS Mix 1 ppm in nitrogen, and SPEX Certi-Prep CAN-TERP-MIX2 100 ug/mL in methanol. The amount of gas standards injected ranged from 0.5 nL to 3 nL. Gas and liquid standards were injected separately into the sorbent tubes for calibration. Lower limit of detection (LLOD) and upper limit of detection (ULOD) were determined for each VOC and technique (Supplementary Tables S1–S2). To adjust for highly concentrated samples, the inlet was split with various ratios, to dilute samples. When standards were run without the split, then sample concentrations were multiplied by the inverse dilution factor or multiplier (M). If the sample concentration was above the ULOD, it was replaced with the ULOD value. Non-detects were labeled (ND).

### 2.6. Data Processing

Following HS-GC-FID and TD-GC-MS analysis, gas phase VOC concentrations ($C_{HS,g}\;or\;C_{TD,g}$) were calculated from peak area to aqueous concentration, $C_{HS,aq}$ or gas amount (nL), respectively, and then converted to parts per billion (ppb) by volume in the original compost air sample (Equations 1 and 2). Variables include $V_{aq}$, the volume of the water used in the impinger (0.005 L), $d_{l}$, the liquid density of the VOC (as standards were prepared from liquid phase by volume), the molar volume of a gas at 25°C and 1 atm, or 24.45 nL/nmole, the molecular weight of the VOC in ng/nmole, the volume of air pumped through the impinger or sorbent tube ($V_{air}$), the inverse dilution factor, M. For eight analytes in Table 3, the total concentration ($C_{Tot}$) was calculated by summing both $C_{HS,g}\left(ppb\right)$ and $C_{TD,g}\left(ppb\right)$, as shown in Equation (3). This was also used to calculate percent recovery of the impinger (see next section).


(1) $$C_{HS,g}\left(ppb\right)={[C}_{HS,aq}\left(\frac{\mu L}{L}\right)-C_{HS,aq,\text{blank}}\left(\frac{\mu L}{L}\right)]∙V_{aq}\left(L\right)∙d_{l}\left(\frac{mg}{\mu L}\right)∙\left(10^{6}\frac{ng}{mg}\right)∙\left(\frac{24.45\;nL/\text{nmole}}{\text{MW ng}/\text{nmole}}\right)∙\left(\frac{1}{V_{air}(L)}\right)$$

(2) $$C_{TD,g}\left(ppb\right)=\frac{\left[nL(VOC)∙M]-nL(\text{blank}\right)}{V_{air}(L)}=\frac{nL}{L}$$

(3) $$(C_{Tot})=C_{HS,g}\left(ppb\right){+C}_{TD,g}\left(ppb\right)$$

### 2.7. Sampling Method Validation and Recovery

Sampling through this dual method was confirmed through percent of analytes captured in the impinger compared to total analyte measured. The concentration of an analyte in the water sample through HS-GC-FID analysis was compared to the same analyte concentration in the air sample plus the water sample, and a percent recovery was calculated using the equation (4) below:

(4) $$\%\text{Recovery}\left(\text{Impinger}\right)=\frac{C_{HS,g}\left(ppb\right)∙100}{C_{HS,g}\left(ppb\right){+C}_{TD,g}\left(ppb\right)}$$

In addition, experiments were run with the use of ice and no ice on the entry of the Teflon tubing into the impinger system, to determine optimal recovery conditions.

## 3. Results and Discussion

119 compounds were analyzed on the TD-GC-MS and 15 compounds were analyzed with the HS-GC-FID. Supplemental Figure S1 displays the chromatograms of the different instrumental methods and select compounds. Full data set is available in Mendeley Data [28].

### 3.1. Sampling Method Validation

The study used a methodology to capture water-soluble VOCs from air in a water impinger trap and prevent escape of those compounds into the air samples. Table 3 depicts the percent recovery for each water-soluble compound measured on both the HS-GC-FID and TD-GC-MS. Percent recovery for water-soluble alcohols ranged between 99–100%, thus the impinger method was effective at capturing these compounds. Likewise, the impinger allowed water-insoluble compounds like terpenes to pass (7 – 48% recovery) and were subsequently caught by the sorbent tube, with percent recovery of 52 – 92% (Table 3). Note, this % recovery is of compounds collected through compost sampling, not spiked or purposefully injected. This represented a distribution or partitioning of the VOCs sampled and analyzed with both analytical systems. The use of ice around the Teflon tubing prior to and around the impinger system showed better recovery of most water-soluble VOCs, and was used in all sampling (Supplemental Figure S2). Most water-soluble VOCs had higher concentrations when the ice was utilized versus not, including methanol, benzaldehyde, acetone, isopropanol, eucalyptol, and guaiacol (Supplemental Figure S2). Ethanol, ethyl acetate, and butanol decreased with the extra ice, but the increased VOCs outweighed these, and thus the ice was utilized in all sampling.

### 3.2. Compost Experiments

**Figure 2:**
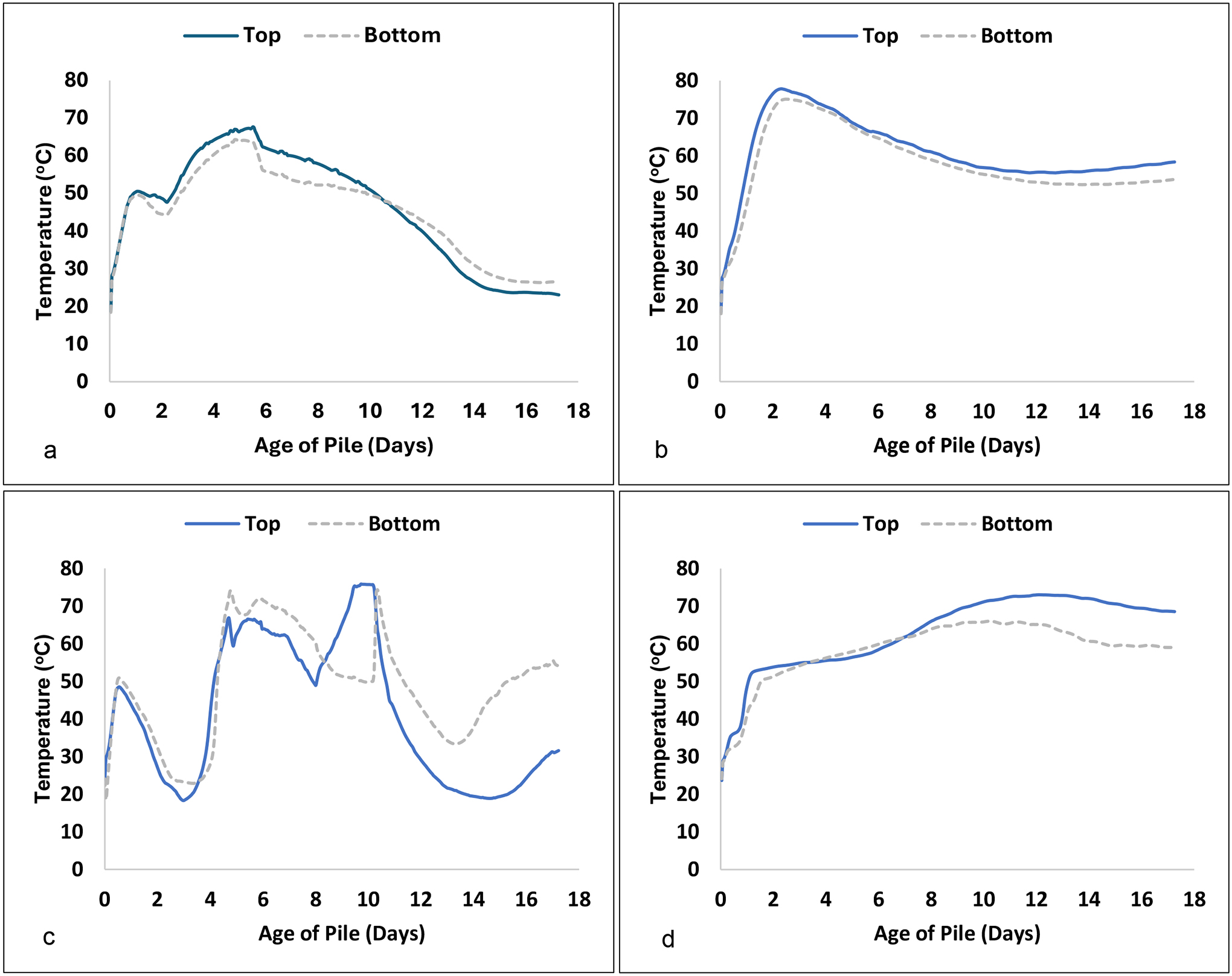
Average temperature profiles of the compost piles studied, from probes located on top and bottom of piles. a) yard waste (YW), low temperature compost b) YW, high temperature compost c) Mixed food/yard waste (FY), low temperature compost d) FY, high temperature.

Temperature profiles from the compost piles are shown in Figure 2. For the yard waste (Exp A), pile 1 peaked at day 5 while pile 2 peaked at day 2. Pile 2 had higher temperatures (up to 77.8°C) sustained over about 10 days compared to pile 1, even though pile 1 started initially at a higher temperature (67.7°C). For the mixed food/yard waste (Exp B), pile 1 temperatures peaked at 5 and 10 days (72.9 °C and 75.8 °C, respectively), while pile 2 peaked at day 12 (73.0°C). On average, yard waste temperatures were 45.8 °C, 59.5 °C, and mixed food/yard waste were 44.9°C, 60.9°C, with pile 1 having a lower temperature than pile 2. For ease of reference, pile 1 will be called low temperature, and pile 2 high temperature, for each experiment.

**Figure 3:**
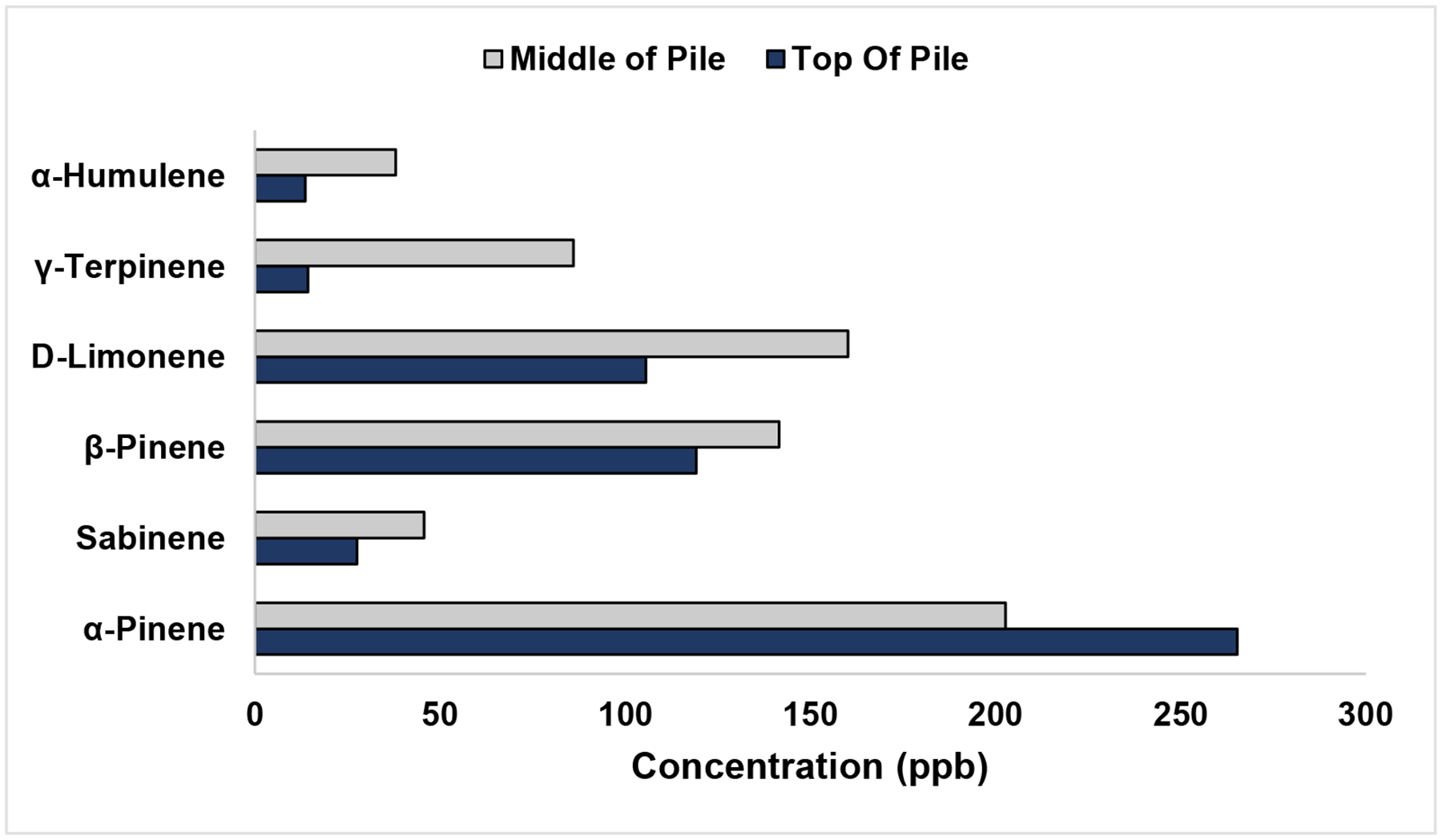
Comparison of emissions at two sampling locations, the middle and top of the piles. The middle of the pile showed overall higher concentrations with the exception of alpha-Pinene. Results are from the Thermal Desorption-Gas Chromatography-Mass Spectrometry (TD-GC-MS) method and yard waste, high temperature experiment (Exp A, YW, Pile 2).

**Figure 4:**
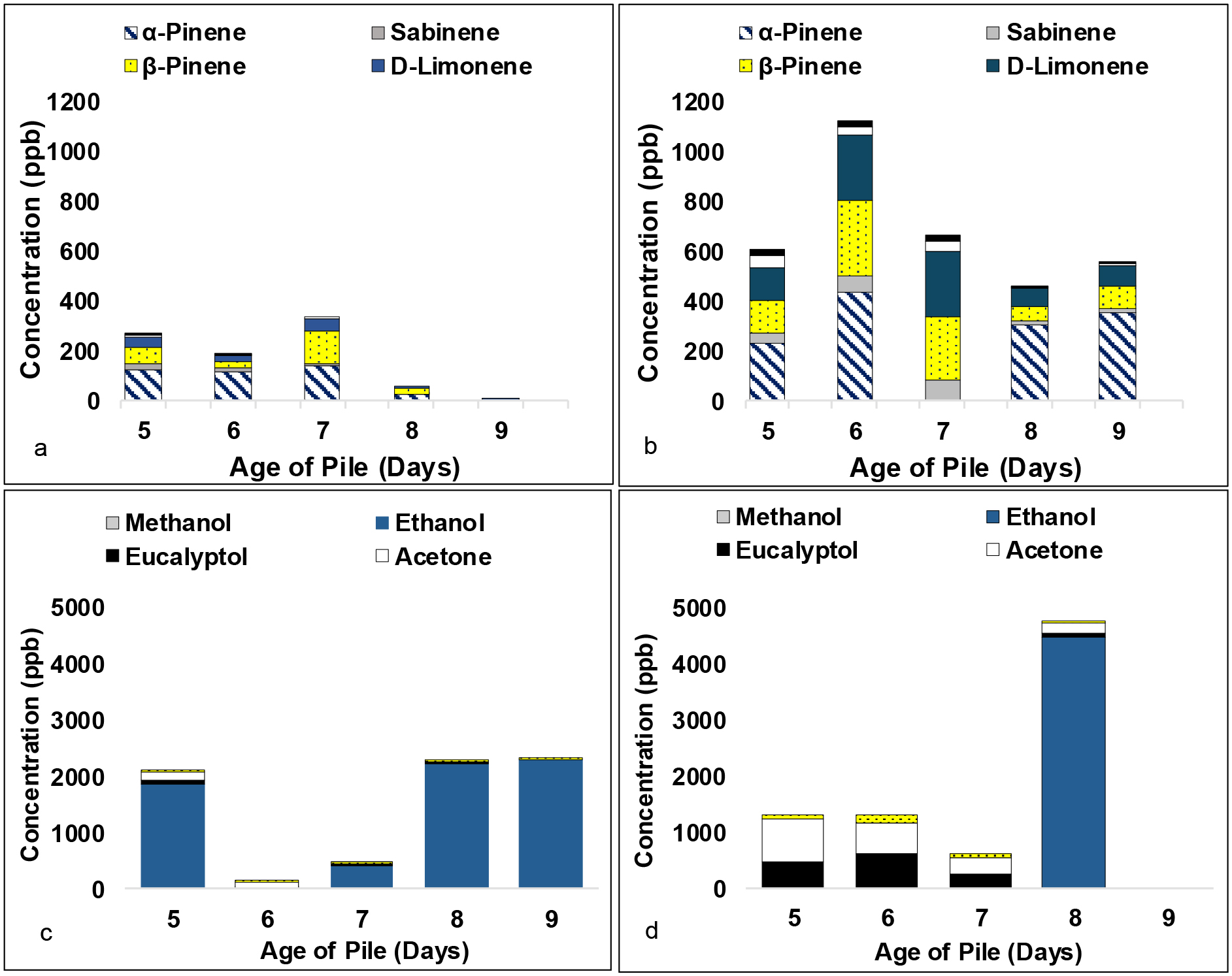
Terpene emissions over time from a) Yard waste (YW), low temperature compost pile and b) YW, high temperature compost pile, via TD-GC-MS analysis (water insoluble). Alcohol and other VOC emissions over five consecutive days from c) YW, Low Temperature compost pile d) YW, High Temperature compost pile, via HS-GC analysis (water soluble).

The position of the sampling on the pile did affect the amounts of VOCs collected (Figure 3), with higher concentrations in the middle of the pile vs the top for terpenes. This was opposite of the chimney effect that was seen by Büyüksönmez [25]. Figure 4 (a,b) displays the terpene emissions ranging significantly higher for the pile with a higher temperature leading to concentrations over 1100 ppb in comparison to 300 ppb found in the low temperature pile. There was a decreasing trend after day 7 for the low temperature pile and day 6 for the high temperature pile. Water-soluble emissions were also captured during this experiment and are displayed in Figure 4 (c,d). Concentrations up to 2000 ppb were emitted from the low temperature pile. Ethanol was the most abundant water-soluble VOC measured in the yard waste, followed by acetone, with emissions still spiking through day nine.

**Figure 5:**
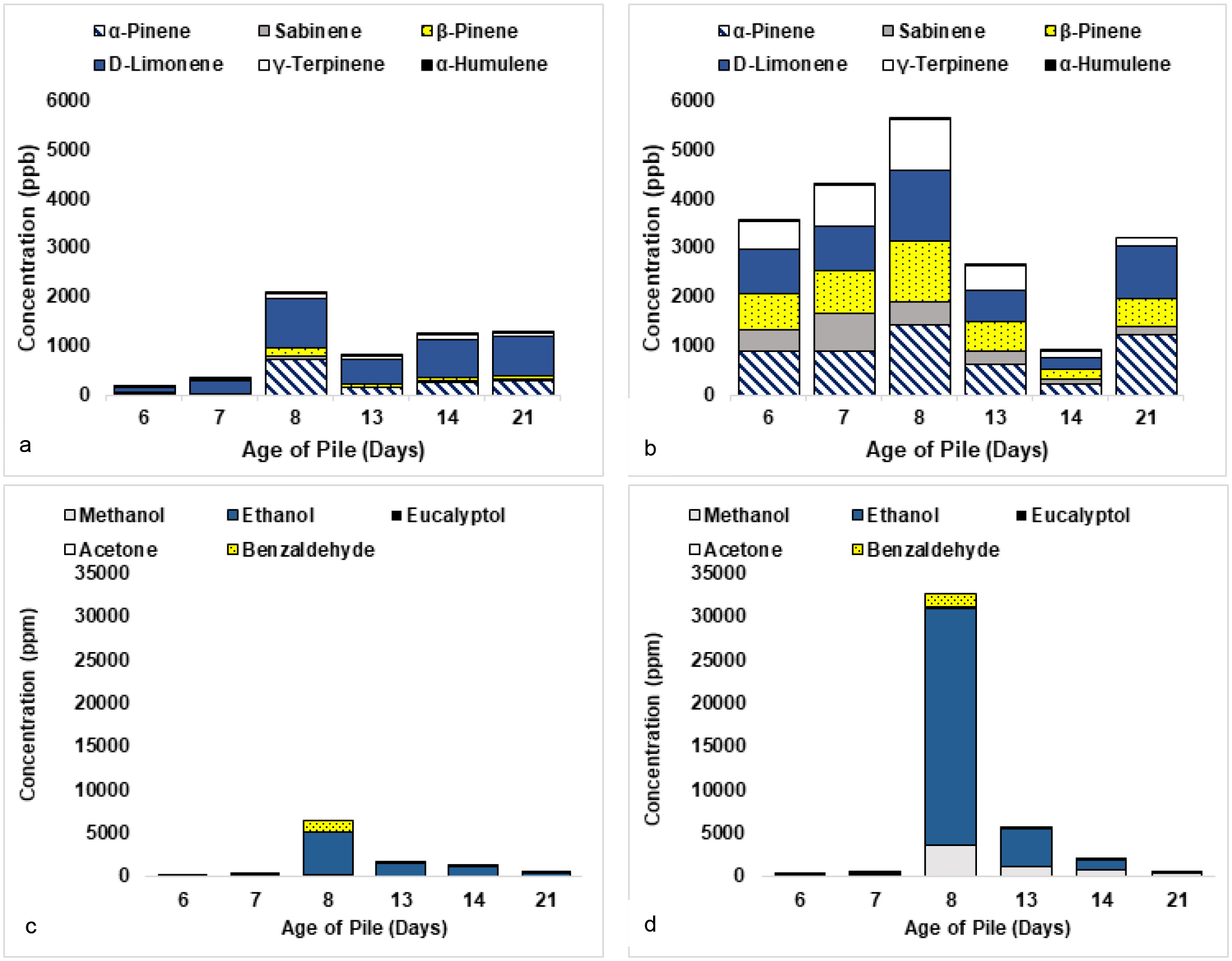
Terpene emissions over time from a) Food/yard waste (FY), Low Temperature compost pile b) FY, High Temp compost pile, via TD-GC-MS analysis (water insoluble). Alcohol and VOC emissions over time from c) FY, Low Temperature compost pile d) FY, High Temperature compost pile, via HS-GC analysis (water soluble).

In experiment B, the composition within the piles changed from strictly yard waste (YW) to a mixture of food and yard waste (FY). The terpenes were seen at higher levels compared to the yard waste compost piles with concentrations ranging up to 2000 ppb for the low temperature pile and 5500 ppb for the high temperature pile (Figure 5 a, b). Both piles peaked in terpene concentrations on day eight, but had a moderate increase on day 21, or towards the end of the experiment. The mixed food/yard waste piles displayed much higher levels of alcohols ranging upwards of 32,000 ppm for the high temperature pile and over 7000 ppb for the low temperature pile (Figure 5 c, d). Ethanol was again the most abundant compound in these emissions. A spike of emissions was seen on day eight in both the low and high temperature piles.

**Figure 6.**
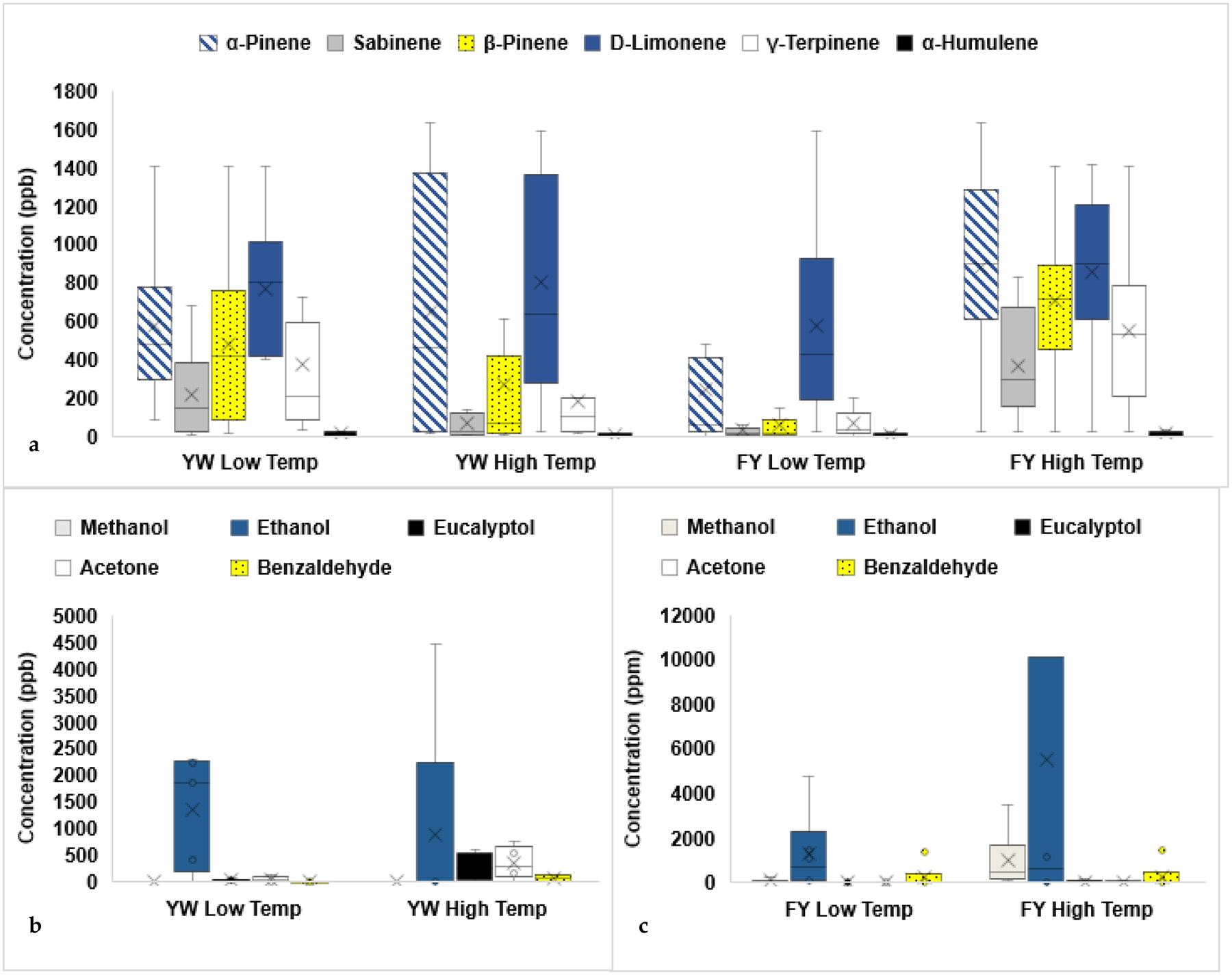
Range in emissions of alcohols, terpenes, and other VOC emissions among different types of compost over the course of the study a) via TD-GC-MS analysis (water insoluble); b,c) via HS-GC analysis (water soluble)

A comparison of the emissions from each pile type is shown in Figure 6. For overall terpene emissions higher levels were seen in the YW pile with concentrations as high as 1600 ppb in comparison to 1250 ppb for FY piles (Figure 6a). Alcohols were seen to have a much higher emission with the FY piles with concentrations reaching 10,000 ppm while the YW pile reached 2100 ppb (Figure 6 b,c).

The composition, temperature, and age of the pile displayed a variety of trends in terms of the VOC emissions. Mixed food/yard (FY) waste especially showed higher levels of ethanol compared to the yard waste alone, with ppm vs. ppb levels. The age of the piles had an overall decreasing trend of emissions once peak emissions were reached with alpha-Pinene and ethanol being the most abundant compounds throughout the experiments. Temperature of the compost pile influenced alcohol and terpene emissions with the higher temperature piles leading to an increase in emissions compared to the lower temperature piles. Hazardous air pollutants were not generally elevated except methanol and acetone (maximum observed values were 3812 ppm and 98 ppm, respectively). Methanol exceeded the permissible exposure limit for 8-hour time weighted average (PEL-TWA) of 200 ppm [21]. Since these observations were inside the piles, and not in the surrounding environment, direct health risk was not calculated, but the composting did generate these hazardous gases.

### 3.3. Comparison to Other Studies

A couple other similar studies measured the flux of various VOCs using similar GC-MS methods. These are summarized in Table 4. Büyüksönmez and Evans [26] measured terpenes in green waste (grass, wood and prunings) using coconut shell sorbent material and GC-MS analysis. Alpha-Pinene was the main contributor of compost emissions, ranging from 10–153 mg/kg dry weight of compost. They detected five other major terpenes: D-Limonene, beta-Pinene, beta-myrcene, 3-carene, and camphene. The emissions of terpenes over time from composting was fairly constant from a couple of days old to over 40 days old. The current study also detected D-Limonene and beta-Pinene in yard waste, with D-Limonene also increasing in mixed food/yard waste. A second study by Büyüksönmez [25] utilized SCAQMD Method 25.3 and a Tenax sorbent trap, to convert VOCs to total non-methane/ethane VOC signal by oxidizing to carbon dioxide and then methane. The total VOCs were measured via FID-GC in green and food waste, with food waste having more total emissions. More VOC emissions were observed in mixed food/yard waste compared to green/yard waste in the current study, measuring speciated, not total VOCs.

Kumar et al [9] utilized a similar analysis to the current study, measuring speciated VOCs in green waste using canisters and GC-MS, as well as measuring the water trap condensate via GC-MS. Their results indicate alcohols as the major contributor of VOC flux, followed by a lesser extent of acids and biogenic VOCs. The current study shows alcohols up to ten times more concentrated than terpenes in yard waste emissions, and even an order of magnitude larger for mixed food/yard waste. The current study is an important comparison of both the method and results of Kumar et al. [9] with a focus on the internal concentrations of the compost pile rather than emitted fluxes.

Gonzalez et al. [32] studied greenhouse gases and speciated VOCs using air sampling bags and sorbent tubes on benchtop sewage sludge-based compost. They used TD-GC-MS analysis and observed terpenes, especially alpha-Pinene in the compost emissions, up to 13,299 ppb on day 2, with a large drop to 12.8 ppb on day 11 [32]. Eucalyptol was also observed almost as high on day 2, at 13,604 ppb [12]. Comparatively, less Pinene and eucalyptol in both YW and FY compost were observed in the current study, with YW releasing more of these than FY, with measurable levels even up to day 21 (Figure 4, 5).

Biasioli et al. [33] measured speciated VOCs, including dimethyl sulfide, methanethiol, acetic acid and acetaldehyde, ranging from 10–200 ppb in compost piles, using proton transfer reaction-mass spectrometry (PTR-MS). They utilized air sampling bags to collect the compost, followed by the PTR-MS analysis. The current study observed values higher than 200 ppb for many VOCs, but only observed 0.7 ppb dimethyl sulfide, for example.

### 3.4. Advantages and Limitations

The sampling and analytical techniques of this study show effective capture of both water-soluble and insoluble VOCs in compost pile emissions. Alcohols in particular are 99–100% recovered in the aqueous impinger. Advantages of this method are speciated VOC characterization, decreased exposure of sensitive equipment to humidity, the timeliness and simplicity of the sampling method and the lower cost compared to canisters and on-line sampling/analysis. A disadvantage of the chromatographic analysis is that it can be time-consuming. Limitations of the data presented here include the unique compost piles built at WSU CF, which may not represent all types of yard and food/yard waste compost. Daily sampling of compost piles was not completed due to budgetary and time constraints. Seasonal differences were not accounted for, as the internal pile, not external was sampled. Overall, the characterization was successful at comparing composition, temperature and age factors that can influence compost emissions, as well as verifying the dual-sampling and analytical method.

## 4. Conclusions

The developed methodology which combined water and air analysis of VOC emissions from compost was successful. Using a cold impinger apparatus, water-soluble compounds were effectively captured with a high percent recovery between 99–100%. This technique allowed for removal of moisture interference in subsequent sorbent tube capture of the remaining water insoluble VOCs.

Two types of compost were analyzed for VOCs (yard waste and food/yard waste), to determine differences in composition and generation of VOCs. Both types emitted a variety of VOCs including terpenes (alpha and beta-Pinene, D-Limonene, gamma-terpinene, sabinene, and alpha-humulene) and alcohols (methanol, ethanol, eucalyptol, acetone, benzaldehyde). Food/yard waste compost produced ethanol up to 27,400 ppm. The highest emissions seen in the timed trials were alpha-Pinene and D-Limonene of the terpenes, and ethanol and methanol of the alcohols, both between days 7–8. Overall, higher emissions were seen at higher temperatures. Composition of the pile influenced the emissions seen with more alcohols produced in the mixed food/yard waste versus yard waste, and terpenes were more present in the yard waste pile. Location of sampling in the pile also affected VOCs, with more concentrated VOCs towards the middle (versus the top) of the pile.

Applications of this study include air pollution monitoring, VOC emission factors, and regulatory standards. Food/yard waste for example, had elevated alcohol emissions within the pile. These emissions can be remediated by use of biofilters, control of temperature or aeration, and/or other variables. In addition, VOC speciation of compost emissions can be utilized in exposure assessment of compost facility workers and nearby residential communities, and subsequent health risks from compost emissions.

## Supplementary Material

supplement berger et al

**Supplementary Materials:** The following supporting information can be downloaded at: www.mdpi.com/xxx/s1, Berger et al. Supplement.pdf

## Figures and Tables

**Table 1.** Compost pile conditions

Experiment	Pile Build Date/Time (PDT)	Feed Stock/Composition	Pile 1 Temperature Profile	Pile 2 Temperature Profile	Pile 1 Aeration	Pile 2 Aeration	Pile cover
A	7/13/2022 12:00	Yard Waste (1)	Low	High	Negative	Negative	No
B	8/26/2022 15:00	Mixed Food/Yard Waste (2,3)	Low	High	Negative	Negative	Yes, screened fines from A piles

1 Whitman County ground green waste, ground in summer, and remnants of the ground pile 1 month later.

2 New Whitman County, summer collected green waste was ground by Cannon Hill at Whitman County Transfer site in August 2022 and trucked by WSU to the WSU Compost Yard

3 Includes ground wood waste material from Asotin landfill

**Table 2.** Summary of Dual Sampling and Analytical Methods

Target Compounds	Water-insoluble VOCs (Hydrocarbons, Terpenes)	Water-soluble VOCs (Alcohols, Aldehydes, Ketones)
Sample Type	Impinger (aqueous)	Dual Sorbent Tube (gas phase)
Instrument	Agilent TD-GC-MS	Agilent HS-GC-FID
Carrier Gas	Ultra-high purity Helium, 1.5 mL/min	Ultra-high purity helium, 1 mL/min
Split Ratio	Varied (split-less to 1:14.3)	Varied 1:2 -1:100
Column	Agilent DB-624, 60 meters, 0.32 mm ID, 1.8 μm thickness	J&W DB-624, 30 meters, 0.32 mm ID, 1.8 μm thickness
Temperature (°C) Programming	**TD**- Pre-purge 2 min, Cold trap −5°C Tube desorption: 50°C for 5 min then 200°C for 10 min Cold trap desorption 250°C for 10 min **GC-** 40°C hold 2 min Ramp1- 40°C-195°C Ramp2- 195°C-250°C, hold 2 min **MS-** 230°C source 150°C quadruple	**HS**- 70°C check these 80°C 200°C transfer line **GC**- 40°C to 195°C Ramp-10°C/min **FID**- 250°C
Detector	Quadruple Electron Ionization Mass Spectrometer 45amu/z-300amu/z Voltage=1801 volts	Flame Ionization Detector with ultra-high purity Hydrogen/Zero Air fuel

**Table 3:** Example distribution of overlapping compounds between the aqueous (HS-GC-FID) and gas (TD-GC-MS) phases and techniques, as well as mean percent recovery of the impinger (See Equation 4). Total concentration reflects the sum of both phases.

Compound	HS-GC-FID Concentration (ppb)	TD-GC-MS Concentration (ppb)	Total Concentration (ppb)	Percent Recovery (%) Impinger	Mean Percent Recovery (%) Impinger
Ethanol	548080	2155	550235	99	99.0
Guaiacol	66950	0	66950	100	100.0
Eucalyptol	50482	19	50501	99	99.9
1-Butanol	549	0	549	100	100.0
Isopropanol	35052	130	35182	99	99.5
D-Limonene	1413	89863	91276	1.5	48.3
alpha-Pinene	208	1413	1621	12.8	7.7
beta-Pinene	46	413	459	10.0	31.6

**Table 4:** Comparison of current study with other VOC compost studies

Study	Compost Type	Sampling/Analytical Technique	Gases measured	Observations (Maximum or ranges)
Current study	Yard and food/yard waste	Impinger Water Trap/HS-GC-FID, Tenax sorbent/TD-GC-MS	Methanol Ethanol Alpha-Pinene Beta-Pinene D-Limonene	3800 ppm 27,400 ppm 1638 ppb 1637 ppb 1750 ppb
Büyüksönmez and Evans [26]	Green waste	Coconut shell sorbent/GC-MS	Alpha-Pinene Beta-Pinene D-Limonene	10–153 mg/kg 1–49 mg/kg 0.1–58 mg/kg
Büyüksönmez [25]	Green and food waste	SCAQMD Method 25.3/Tenax sorbent/GC-FID	Total Non-methane/ethane VOC	2–40 ppm
Kumar et al. [9]	Green waste	Water trap, Canisters, GC-MS	Speciated VOCs Alcohols Terpenes, Acids	2.6–13 mg/m^2^/min
Gonzalez et al. [32]	Benchtop sewage-sludge	Air bags/sorbent tubes/TD-GC-MS	Alpha-Pinene Beta-Pinene D-Limonene Eucalyptol	13299 ppb 6390 ppb 5490 ppb 13605 ppb
Biasioli et al. [33]	Compost piles with and without biofilter	Air bags, Proton transfer reaction- mass spectrometry	Dimethyl sulfide methanethiol acetic acid acetaldehyde	20 ppb 10 ppb 100 ppb 200 ppb

## Data Availability

The original data presented in the study are openly available in Mendeley Data at doi: 10.17632/9t9d6f4kjc.1

## Abbreviations

The following abbreviations are used in this manuscript:

VOC: Volatile Organic Compound
TD-GC-MS: Thermal Desorption-Gas Chromatography-Mass Spectrometry
HS-GC-FID: Headspace-Gas Chromatography-Flame Ionization Detection
US EPA: United States Environmental Protection Agency
SCAQMD: South Coast Air Quality Management District
YW: Yard waste
FY: Combined food/yard waste
EPA: Environmental Protection Agency
OSHA: Occupational Safety and Health Administration
PEL: Permissible Exposure Limit
TWA: Time Weighted Average
WSU CF: Washington State University Compost Facility
ppm: Parts per million
ppb: Parts per billion
