# Supplementary material for: Analysis of Volatile Organic Compounds from Compost
Source: Atmosphere (Basel). Author manuscript; Available in PMC 2026 Jun 16. (PMC13268598; doi:10.3390/atmos16050591)
Supplement: supplement berger et al [file NIHMS2130594-supplement-supplement_berger_et_al.docx]

Analysis of Volatile Organic Compounds from Compost

Shastine K. Berger^1^, Rosario C. Morales^1^, Katherine McCown^1^, Kylie Wilson^1^, B. Thomas Jobson^2^ and Nancy A.C Johnston^*1^

|  |
| --- |

^1^ Physical, Life, Movement, & Sport Sciences Division, Lewis-Clark State College, Lewiston, Idaho

^2^ Department of Civil and Environmental Engineering, Washington State University, Pullman, Washington

***** Correspondence: najohnston@lcsc.edu; Tel.: (+1-208-792-2233)

**Supplement**

**Table of Contents:**

| Page | Item | Description |
| --- | --- | --- |
| 2 | Table S1 | Overview of Volatile Organic Compound (VOC) information for Headspace-Gas Chromatography analytical method. |
| 3 | Table S2 | Overview of Volatile Organic Compound (VOC) information for Thermal Desorption-Gas Chromatography-Mass Spectrometry analytical method. |
| 6 | Figure S1 | Annotated chromatograms of alcohol standard from the HS-GC-FID and terpene standard from the TD-GC-MS. |
| 7 | Figure S2 | Duplicate trials conducted with Teflon tubing in no ice, and ice prior to the impinger in ice bath. |
|  |  |  |

Table S1: Overview of Volatile Organic Compound (VOC) information for Headspace-Gas Chromatography (HS-GC-FID) analytical method.

| *HS-GC*  *Ref#* | *TD-GC-MS*  *Ref#* | *Compound* | *Formula* | *MW* | *R.T.* | *Response factor* | *R^2^* | *LOD* | *ULOD* |
| --- | --- | --- | --- | --- | --- | --- | --- | --- | --- |
| 1 | NA | Methanol | CH_3_OH | 32.04 | 2.60 | 1.28E+05 | 0.998 | 0.01 | 39.55 |
| 2 | 15 | Ethanol | C₂H₆O | 46.07 | 3.40 | 4.72E+04 | 0.999 | 0.02 | 39.45 |
| 3 | NA | Acetone | C_3_H_6_O | 58.08 | 3.76 | 2.25E+05 | 0.999 | 0.02 | 39.2 |
| 4 | 19 | Isopropanol | C_3_H_8_O | 60.10 | 4.03 | 5.15E+04 | 0.999 | 0.06 | 39.3 |
| 5 | NA | Ethyl acetate | C_4_H_8_O_2_ | 88.11 | 5.70 | 5.71E+05 | 0.999 | 0.03 | 45.1 |
| 6 | 40 | Butanol | C₄H₁₀O | 74.12 | 7.10 | 1.44E+05 | 0.999 | 0.04 | 40.5 |
| 7 | NA | Acetaldehyde | C_2_H_4_O | 44.05 | 8.86 | 7.46E+04 | 0.999 | 0.02 | 39.4 |
| 8 | NA | 1-Pentanol | C_5_H_12_O | 88.15 | 9.16 | 2.16E+05 | 0.999 | 0.04 | 40.7 |
| 9 | 84 | Eucalyptol | C_10_H_18_O | 154.25 | 11.69 | 6.21E+03 | 0.958 | 0.08 | 46.05 |
| 10 | 77 | Beta-Pinene | C_10_H_16_ | 136.23 | 12.60 | 1.32E+04 | 0.937 | 0.08 | 43.6 |
| 11 | NA | Benzaldehyde | C₇H₆O | 106.12 | 13.08 | 6.72E+02 | 0.963 | 0.01 | 52.2 |
| 12 | 80 | D-Limonene | C_10_H_16_ | 136.24 | 13.40 | 1.49E+04 | 0.998 | 0.07 | 42.05 |
| 13 | 69 | alpha-Pinene | C_10_H_16_ | 136.24 | 13.65 | 4.69E+05 | 0.988 | 0.02 | 42.9 |
| 14 | 96 | Guaiacol | C_7_H_8_O_2_ | 124.14 | 15.30 | 2.68E+04 | 0.999 | 0.01 | 56.45 |
| 15 | NA | Eugenol | C_10_H_12_O_2_ | 164.20 | 19.54 | 1.35E+04 | 0.988 | 0.003 | 53.35 |

Units and abbreviations: Molecular Weight (MW, amu), Quantitation Ion (QIon, m/z), Retention time (RT, min), Slope (1/ng), Limit of detection (LOD, mg/L).

**Table S2:** Overview of Volatile Organic Compound (VOC) information for Thermal Desorption-Gas Chromatography-Mass Spectrometry (TD-GC-MS) analytical method.

| *Ref #* | *Compound* | *Formula* | *MW* | *QIon* | *RT* | *Response Factor* | *R^2^* | *LLOD* | *ULOD* |
| --- | --- | --- | --- | --- | --- | --- | --- | --- | --- |
| 1 | Dichlorodifluoromethane | CCl_2_F_2_ | 120.91 | 85 | 4.14 | 1.60E+06 | 0.999 | 0.002 | 1.5 |
| 2 | Ethane, 1,2-dichloro-1,1,2,2-tetrafluoro | C_2_Cl_2_F_4_ | 170.92 | 85 | 4.40 | 1.17E+06 | 0.999 | 0.002 | 1.5 |
| 3 | Chloromethane | CH_3_Cl | 50.49 | 50 | 4.49 | 3.78E+05 | 0.998 | NA | 1.5 |
| 4 | 1-Butene | C_4_H_8_ | 56.11 | 56 | 4.68 | 2.56E+05 | 0.984 | 0.027 | 1.5 |
| 5 | Ethene, chloro- | C_4_H_4_Cl_4_ | 193.90 | 62 | 4.72 | 5.85E+05 | 0.999 | 0.003 | 1.5 |
| 6 | 2-Butene (Z), cis | C_4_H_8_ | 56.11 | 56 | 4.86 | 3.36E+05 | 0.995 | 0.009 | 1.5 |
| 7 | 2-Butene (E), trans | C_4_H_8_ | 56.11 | 56 | 5.04 | 4.13E+05 | 0.988 | 0.008 | 1.5 |
| 8 | Bromomethane | CH_3_Br | 94.94 | 94 | 5.32 | 1.71E+04 | 0.981 | NA | 1.5 |
| 9 | Ethyl Chloride | C_2_H_5_Cl | 64.51 | 64 | 5.46 | 3.00E+05 | 0.997 | 0.004 | 1.5 |
| 10 | Butane, 2-methyl | C_5_H_12_ | 72.11 | 57 | 5.60 | 3.10E+05 | 0.984 | 0.041 | 1.5 |
| 11 | 1-Pentene | C_5_H_10_ | 70.13 | 55 | 5.87 | 4.53E+05 | 0.990 | 0.006 | 1.5 |
| 12 | Trichloromonofluoromethane | CCl_3_F | 137.37 | 101 | 5.87 | 1.12E+06 | 0.994 | 0.003 | 1.5 |
| 13 | 2-Pentene (Z), cis | C_5_H_10_ | 70.13 | 55 | 6.15 | 9.52E+05 | 0.993 | 0.015 | 1.5 |
| 14 | Isoprene | C_5_H_8_ | 68.12 | 67 | 6.28 | 5.30E+05 | 0.994 | 0.021 | 1.5 |
| 15 | Ethanol | C_2_H_6_O | 46.07 | 46 | 6.29 | *5.863E+04 | 0.992 | 0.074 | 6.3 |
| 16 | Ethene, 1,1-dichloro- | C_2_H_2_Cl_2_ | 96.94 | 101 | 6.59 | 1.07E+06 | 0.999 | 0.003 | 1.5 |
| 17 | Dimethyl Sulfide | C_2_H_6_S | 62.14 | 62 | 6.61 | 5.47E+05 | 0.998 | 0.014 | 5.0 |
| 18 | Butane, 2,2-dimethyl | C_6_H_14_ | 86.18 | 71 | 6.64 | 5.11E+05 | 0.993 | 0.007 | 1.5 |
| 19 | Isopropanol | C_3_H_8_O | 60.10 | 45 | 6.89 | *3.393E+05 | 0.971 | 0.020 | 4.8 |
| 20 | Carbon Disulfide | CS_2_ | 76.15 | 76 | 6.96 | 1.64E+06 | 0.991 | 0.004 | 1.5 |
| 21 | Methylene Chloride | CH_2_Cl_2_ | 84.93 | 84 | 7.17 | 6.37E+05 | 0.998 | 0.003 | 1.5 |
| 22 | Pentane, 2-methyl | C_6_H_14_ | 86.18 | 71 | 7.22 | 5.44E+05 | 0.992 | 0.040 | 1.5 |
| 23 | Ethylene, 1,2-dichloro (E), trans | C_2_H_2_Cl_2_ | 96.94 | 61 | 7.53 | 9.59E+05 | 0.998 | 0.002 | 1.5 |
| 24 | Pentane, 3-methyl | C_6_H_14_ | 86.18 | 57 | 7.55 | 6.98E+05 | 0.985 | 0.035 | 1.5 |
| 25 | 1-Hexene | C_6_H_12_ | 84.16 | 56 | 7.75 | 7.63E+05 | 0.987 | 0.011 | 1.5 |
| 26 | n-Hexane | C_6_H_14_ | 86.18 | 57 | 7.87 | 1.11E+06 | 0.986 | 0.007 | 3.0 |
| 27 | Ethane, 1,1-dichloro- | C_2_H_4_Cl_2_ | 98.96 | 63 | 8.03 | 1.22E+06 | 0.998 | 0.003 | 1.5 |
| 28 | Pentane, 2,4-dimethyl | C_7_H_16_ | 100.20 | 57 | 8.52 | 4.82E+05 | 0.998 | 0.067 | 1.5 |
| 29 | Cyclopentane, methyl- | C_6_H_12_ | 84.16 | 56 | 8.68 | 9.55E+05 | 0.979 | 0.017 | 1.5 |
| 30 | Ethylene, 1,2-dichloro (Z), cis | C_2_H_2_Cl_2_ | 96.94 | 61 | 8.73 | 1.21E+06 | 0.999 | 0.003 | 1.5 |
| 31 | Chloroform | CHCl_3_ | 119.38 | 83 | 9.07 | 1.70E+06 | 0.999 | 0.002 | 1.5 |
| 32 | Hexane, 2-methyl- | C_7_H_16_ | 100.20 | 85 | 9.35 | 8.38E+05 | 0.998 | 0.031 | 1.5 |
| 33 | Ethane, 1,1,1-trichloro- | C_2_H_3_Cl_3_ | 133.40 | 97 | 9.40 | 5.42E+05 | 0.986 | 0.006 | 1.5 |
| 34 | Cyclohexane | C_6_H_12_ | 84.16 | 56 | 9.51 | 8.67E+05 | 0.997 | 0.009 | 3.0 |
| 35 | Hexane, 3-methyl | C_7_H_16_ | 100.20 | 71 | 9.57 | 6.77E+05 | 0.997 | 0.035 | 1.5 |
| 36 | Carbon Tetrachloride | CCl_4_ | 153.82 | 117 | 9.62 | 4.95E+05 | 0.999 | 0.010 | 1.5 |
| 37 | Ethane, 1,2-dichloro- | C_2_H_4_Cl_2_ | 98.96 | 62 | 9.80 | 9.30E+05 | 0.998 | 0.002 | 1.5 |
| 38 | Benzene | C_6_H_6_ | 78.05 | 78 | 9.84 | 2.59E+06 | 0.980 | 0.014 | 3.0 |
| 39 | Heptane | C_7_H_16_ | 100.20 | 71 | 10.08 | 9.12E+05 | 0.974 | 0.006 | 3.0 |
| 40 | 1-Butanol | C_4_H_10_O | 74.12 | 56 | 10.33 | *2.076E+05 | 0.997 | 0.007 | 4.0 |
| 41 | Trichloroethylene | C_2_HCl_3_ | 131.40 | 130 | 10.60 | 1.52E+06 | 0.999 | 0.004 | 1.5 |
| 42 | Propane, 1,2-dichloro- | C_3_H_6_Cl_2_ | 112.98 | 63 | 10.86 | 8.79E+05 | 0.997 | 0.005 | 1.5 |
| 43 | Cyclohexane, methyl | C_7_H_14_ | 98.19 | 83 | 10.90 | 8.51E+05 | 0.997 | 0.039 | 1.5 |
| 44 | 1,4-Dioxane | C_4_H_8_O_2_ | 88.11 | 88 | 10.99 | 6.87E+05 | 0.998 | 0.006 | 1.5 |
| 45 | Methane, bromodichloro- | CHBrCl_2_ | 163.8 | 83 | 11.17 | 1.78E+06 | 0.998 | 0.004 | 1.5 |
| 46 | Pentane, 2,3,4-trimethyl | C_8_H_18_ | 114.23 | 71 | 11.36 | 1.21E+06 | 0.995 | 0.045 | 1.5 |
| 47 | Heptane, 2-methyl | C_8_H_18_ | 114.23 | 57 | 11.55 | 1.43E+06 | 0.988 | 0.027 | 1.5 |
| 48 | 1-Propene, 1,3-dichloro- | C_3_H_4_Cl_2_ | 110.97 | 75 | 11.73 | 1.57E+06 | 0.999 | 0.005 | 1.5 |
| 49 | Heptane, 3-methyl | C_8_H_18_ | 114.23 | 85 | 11.75 | 1.11E+06 | 0.994 | 0.033 | 1.5 |
| 50 | Methyl Isobutyl Ketone | C_6_H_12_O | 100.16 | 58 | 11.85 | 7.38E+05 | 0.997 | 0.005 | 1.5 |
| 51 | Dimethyl Disulfide | C_2_H_6_S_2_ | 94.20 | 94 | 11.90 | 1.75E+06 | 0.985 | 0.004 | 4.1 |
| 52 | Toluene | C_7_H_8_ | 92.14 | 91 | 12.22 | 3.57E+06 | 0.979 | 0.005 | 3.0 |
| 53 | Octane | C_8_H_18_ | 114.26 | 85 | 12.28 | 9.23E+05 | 0.991 | 0.030 | 1.5 |
| 54 | 1-Propene, 2,3-dichloro- | C_3_H_4_Cl_2_ | 110.97 | 75 | 12.41 | 1.34E+06 | 0.999 | 0.004 | 1.5 |
| 55 | Ethane, 1,1,2-trichloro- | C_2_H_3_Cl_3_ | 133.40 | 97 | 12.68 | 1.19E+06 | 0.997 | 0.005 | 1.5 |
| 56 | 2-Hexanone | C_6_H_12_O | 100.16 | 58 | 12.94 | 9.51E+05 | 0.995 | 0.006 | 1.5 |
| 57 | Tetrachloroethylene | C_2_Cl_4_ | 165.83 | 166 | 13.00 | 2.06E+06 | 0.997 | 0.005 | 1.5 |
| 58 | Methane, dibromochloro- | CHBr_2_Cl | 208.28 | 129 | 13.27 | 2.15E+06 | 0.998 | 0.005 | 1.5 |
| 59 | Ethane, 1,2-dibromo- | C_2_H_4_Br_2_ | 187.86 | 107 | 13.47 | 1.77E+06 | 0.998 | 0.006 | 1.5 |
| 60 | Benzene, chloro- | C_6_H_5_Cl | 112.56 | 112 | 14.15 | 2.88E+06 | 0.997 | 0.007 | 1.5 |
| 61 | Ethylbenzene | C_8_H_10_ | 106.17 | 91 | 14.26 | 4.31E+06 | 0.994 | 0.006 | 3.0 |
| 62 | Nonane | C_9_H_20_ | 128.25 | 57 | 14.36 | 1.23E+06 | 0.969 | 0.041 | 1.5 |
| 63 | Furfural | C_5_H_4_O_2_ | 96.08 | 96 | 14.37 | *3.510E+05 | 0.993 | 0.005 | 4.4 |
| 64 | Xylene ,m,p- | C_8_H_10_ | 106.16 | 91 | 14.42 | 3.04E+06 | 0.995 | 0.078 | 6.0 |
| 65 | Styrene | C_8_H_8_ | 104.06 | 104 | 15.00 | 2.67E+06 | 0.993 | 0.007 | 3.0 |
| 66 | Xylene,o- | C_8_H_10_ | 106.16 | 91 | 15.00 | 3.22E+06 | 0.990 | 0.038 | 3.0 |
| 67 | Methane, tribromo- | CHBr_3_ | 252.73 | 173 | 15.30 | 2.19E+06 | 0.992 | 0.006 | 1.5 |
| 68 | Benzene, (1-methylethyl) | C_9_H_12_ | 120.19 | 105 | 15.50 | 3.44E+06 | 0.974 | 0.040 | 1.5 |
| 69 | alpha-Pinene | C_10_H_16_ | 136.23 | 93 | 15.54 | *5.214E+05 | 0.997 | 0.016 | 2.7 |
| 70 | Ethane, 1,1,2,2-tetrachloro- | C_2_H_2_Cl_4_ | 167.85 | 83 | 15.86 | 2.30E+06 | 0.977 | 0.010 | 1.5 |
| 71 | Benzene, propyl- | C_9_H_12_ | 120.20 | 91 | 16.10 | 3.73E+06 | 0.964 | 0.048 | 1.5 |
| 72 | Benzene, 1-ethyl-3-methyl | C_9_H_12_ | 120.19 | 105 | 16.22 | 3.11E+06 | 0.960 | 0.063 | 1.5 |
| 73 | Benzene, 1-ethyl-4-methyl- | C_9_H_12_ | 120.19 | 105 | 16.26 | 4.90E+06 | 0.981 | 0.040 | 3.0 |
| 74 | Decane | C_10_H_22_ | 142.28 | 57 | 16.27 | 1.38E+06 | 0.960 | 0.061 | 1.5 |
| 75 | Mesitylene | C_9_H_12_ | 120.19 | 105 | 16.34 | 3.79E+06 | 0.982 | 0.013 | 3.0 |
| 76 | Sabinene | C_10_H_16_ | 136.23 | 93 | 16.36 | *5.188E+05 | 0.996 | 0.019 | 2.7 |
| 77 | beta-Pinene | C_10_H_16_ | 136.23 | 93 | 16.53 | *5.670E+05 | 0.994 | 0.033 | 2.7 |
| 78 | Benzene, 1-ethyl-2-methyl | C_9_H_12_ | 120.19 | 105 | 16.66 | 2.98E+06 | 0.964 | 0.053 | 1.5 |
| 79 | Benzene, 1,2,4-trimethyl- | C_9_H_12_ | 120.19 | 105 | 16.91 | 3.95E+06 | 0.980 | 0.012 | 3.0 |
| 80 | D-Limonene | C_10_H_16_ | 136.23 | 93 | 17.32 | *2.119E+05 | 0.997 | 0.031 | 2.7 |
| 81 | Benzene, 1,3-dichloro- | C_6_H_4_Cl_2_ | 147.00 | 146 | 17.39 | 2.71E+06 | 0.981 | 0.018 | 1.5 |
| 82 | Benzene, 1,2-dichloro- | C_6_H_4_Cl_2_ | 147.00 | 146 | 17.51 | 2.71E+06 | 0.979 | 0.021 | 1.5 |
| 83 | Benzene, 1,2,3-trimethyl | C_9_H_12_ | 120.19 | 105 | 17.56 | 2.46E+06 | 0.966 | 0.046 | 1.5 |
| 84 | Eucalyptol | C_10_H_18_O | 154.25 | 81 | 17.60 | *1.355E+05 | 0.990 | 0.003 | 2.2 |
| 85 | Benzyl Chloride | C_7_H_7_Cl | 126.58 | 91 | 17.67 | 2.94E+06 | 0.984 | 0.013 | 1.5 |
| 86 | Phenol, 2-chloro- | C_6_H_5_ClO | 128.55 | 128 | 17.68 | *4.510E+05 | 0.983 | 0.004 | 2.9 |
| 87 | gamma-Terpinene | C_10_H_16_ | 136.23 | 93 | 17.79 | *4.176E+05 | 0.996 | 0.029 | 2.7 |
| 88 | Benzene, 1,3-diethyl | C_10_H_14_ | 134.22 | 119 | 17.80 | 1.93E+06 | 0.974 | 0.044 | 1.5 |
| 89 | Phenol | C_6_H_6_O | 94.11 | 94 | 17.88 | *4.384E+05 | 0.990 | 0.010 | 3.9 |
| 90 | Benzene, 1,4-diethyl | C_10_H_14_ | 134.22 | 119 | 17.94 | 1.96E+06 | 0.973 | 0.048 | 1.5 |
| 91 | Undecane | C_11_H_24_ | 156.31 | 57 | 18.05 | 1.55E+06 | 0.985 | 0.078 | 1.5 |
| 92 | Benzene, 1,4-dichloro- | C_6_H_4_Cl_2_ | 147.00 | 146 | 18.10 | 2.54E+06 | 0.974 | 0.017 | 1.5 |
| 93 | alpha-Terpinolene | C_10_H_16_ | 136.23 | 93 | 18.39 | *2.536E+05 | 0.997 | 0.023 | 2.7 |
| 94 | Sabinene hydrate | C_10_H_18_O | 154.25 | 93 | 18.63 | *2.369E+05 | 0.995 | 0.086 | 2.4 |
| 95 | L-Fenchone | C_10_H_16_O | 152.23 | 81 | 19.27 | *5.824E+05 | 0.994 | 0.018 | 2.4 |
| 96 | Guaiacol | C_7_H_8_O_2_ | 124.14 | 109 | 19.39 | *3.296E+05 | 0.982 | 0.008 | 3.0 |
| 97 | Fenchol | C_10_H_18_O | 154.25 | 81 | 19.86 | *2.857E+05 | 0.994 | 0.020 | 2.4 |
| 98 | Dodecane | C_12_H_26_ | 170.33 | 57 | 19.97 | 1.64E+06 | 1.000 | 0.055 | 1.5 |
| 99 | Phenol, 2-nitro- | C_6_H_5_NO_3_ | 139.11 | 139 | 20.57 | *1.422E+05 | 0.997 | 0.002 | 2.6 |
| 100 | Camphor | C_10_H_16_O | 152.23 | 95 | 20.85 | *5.221E+05 | 0.993 | 0.016 | 2.4 |
| 101 | Phenol, 2,4-dimethyl- | C_8_H_10_O | 122.16 | 122 | 20.97 | *3.356E+05 | 0.972 | 0.006 | 3.0 |
| 102 | Benzene, 1,2,4-trichloro- | C_6_H_3_Cl_3_ | 181.45 | 180 | 21.10 | 2.30E+06 | 0.973 | 0.059 | 1.5 |
| 103 | Borneol | C_10_H_18_O | 154.25 | 95 | 21.23 | *1.033E+06 | 0.991 | 0.022 | 2.4 |
| 104 | alpha-Terpineol | C_10_H_18_O | 154.25 | 136 | 21.38 | *7.988E+04 | 0.990 | 0.018 | 2.4 |
| 105 | 1,3-Butadiene, 1,1,2,3,4,4-hexachloro- | C_4_Cl_6_ | 260.76 | 225 | 21.48 | 2.41E+06 | 0.968 | 0.068 | 1.5 |
| 106 | Phenol, 2,4-dichloro- | C_6_H_4_Cl_2_O | 163.00 | 162 | 21.94 | *3.080E+05 | 0.980 | 0.004 | 2.2 |
| 107 | Geraniol | C_10_H_18_O | 154.25 | 69 | 22.57 | *1.634E+05 | 0.984 | 0.050 | 2.4 |
| 108 | Pulegone | C_10_H_16_O | 152.23 | 81 | 22.96 | *2.124E+05 | 0.994 | 0.022 | 2.4 |
| 109 | Phenol, 4-chloro-3-methyl- | C_7_H_7_ClO | 142.58 | 107 | 26.65 | *2.492E+05 | 0.973 | 0.010 | 2.6 |
| 110 | Syringol | C_8_H_10_O_3_ | 154.16 | 154 | 27.09 | *1.840E+05 | 0.968 | 0.014 | 2.4 |
| 111 | Phenol, 2,4,6-trichloro- | C_6_H_3_Cl_3_O | 197.40 | 196 | 28.08 | *2.044E+05 | 0.980 | 0.010 | 1.9 |
| 112 | alpha-Cedrene | C_15_H_24_ | 204.35 | 119 | 28.61 | *5.188E+05 | 0.996 | 0.013 | 1.8 |

Units are as follows: Molecular Weight (MW, amu), Quantitation Ion (QIon, m/z), Retention time (RT, min), Response Factor (1/nL), Lower Limit of detection (LLOD, ppbv), Upper limit of detection (ULOD, ppbv).

*Units for the response factor are 1/ng for select compounds from liquid standards


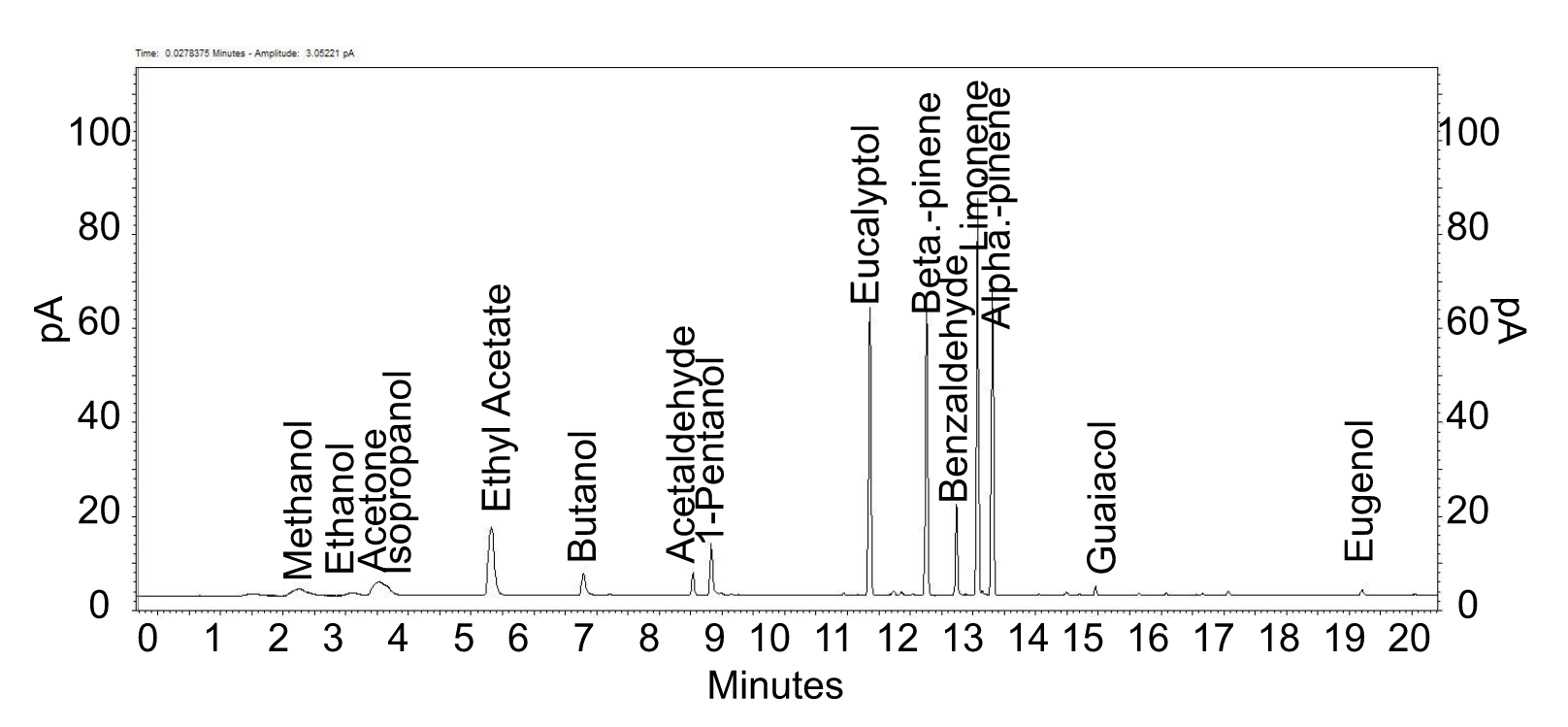


a)


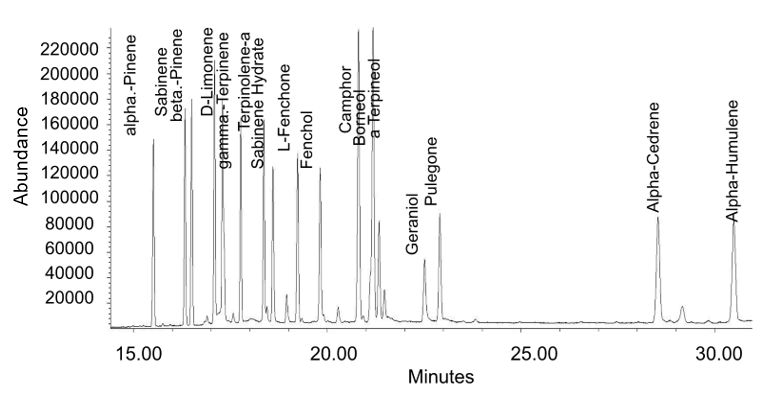


b)

Figure S1: Annotated chromatograms of a) alcohol standard from the HS-GC-FID analysis, with picoAmp (pA) units for FID detector signal versus time in minutes b) terpene standard from the TD-GC-MS analysis, with abundance (unitless) for mass-spectrometer signal versus time in minutes. Instrument conditions: DB 624 column, 32 mm, 1.8 µm a) 30 m column, He carrier 1 mL/min, Flame ionization detector (FID) b) 60 m column, He carrier 1.5 mL/min, Single Quadruple Mass Spectrometer (MS)

Figure S2. Duplicate trials conducted with Teflon tubing in no ice and ice prior to the impinger in ice bath. Results are from the Headspace-Gas Chromatography-Flame Ionization Detector (HS-GC-FID) method. Concentration of gases are in ppb, but note the different scales due to the higher concentrations of methanol, ethanol and benzaldehyde.
